# NMR structure of an acyl-carrier protein from *Borrelia burgdorferi*

**DOI:** 10.1107/S1744309111004386

**Published:** 2011-08-13

**Authors:** Ravi P. Barnwal, Wesley C. Van Voorhis, G. Varani

**Affiliations:** a Department of Chemistry, University of Washington, Seattle, WA 98195, USA; b Seattle Structural Genomics Center for Infectious Disease (SSGCID), USA; c Department of Medicine, University of Washington, Seattle, WA 98195-6423, USA; d Department of Chemistry and Biochemistry, University of Washington, Seattle, WA 98195, USA

**Keywords:** NMR, acyl-carrier proteins, *Borrelia burgdorferi*

## Abstract

Nearly complete resonance assignment and the high-resolution NMR structure of the acyl-carrier protein from *Borrelia burgdorferi*, a target of the Seattle Structural Genomics Center for Infectious Disease (SSGCID) structure-determination pipeline, are reported. This protein was chosen as a potential target for drug-discovery efforts because of its involvement in fatty-acid biosynthesis, an essential metabolic pathway, in bacteria. It was possible to assign >98% of backbone resonances and >92% of side-chain resonances using multidimensional NMR spectroscopy. The NMR structure was determined to a backbone r.m.s.d. of 0.4 Å and contained four α-helices and two 3_10_-helices. A structure-homology search revealed that this protein is highly similar to the acyl-carrier protein from *Aquifex aeolicus*.

## Abstract 2

The high-resolution NMR structure of the acyl-carrier protein from the pathogen *B. burgdorferi* determined to a r.m.s. deviation of 0.4 Å over the protein backbone is reported. The NMR structure was determined using multidimensional NMR spectroscopy and consists of four α-helices and two 3_10_-helices. Structural comparison reveals that this protein is highly similar to the acyl-carrier protein from *A. aeolicus*.

## 1. Biological context

*Borrelia burgdorferi* is a Gram-negative bacterium and a cause of Lyme disease. It was isolated and cultured in the 1980s and was named after its discoverer (Burgdorfer *et al.*, 1982 ▶). Its complete genome sequence was reported in the late 1990s (Fraser *et al.*, 1997 ▶) and provided clues to the role of different genes in the pathogenesis, prevention and treatment of Lyme disease (Guidoboni *et al.*, 2006 ▶). Interestingly, this pathogenic bacterium can survive without iron and this property appears to be an important factor in its survival. We selected an acyl-carrier protein, BobuA.00658.a, from *B. burgdorferi* for structure determination within the SSGCID pipeline. This protein is important in fatty-acid biosynthesis. Because of considerable mechanistic and structural differences from the same processes in eukaryotes, enzymes in this pathway represent attractive antibacterial targets. The acyl-carrier protein is a universal and highly conserved carrier of acyl intermediates during fatty-acid biosynthesis. In yeast, these proteins exist as separate domains within a large multifunctional fatty-acid syntheses polyprotein, whereas in bacteria they are mostly monomeric proteins. These proteins are also cofactors of various primary and secondary pathways, including signaling and production of natural bioactive products. For these reasons, these proteins are interesting drug targets for novel antibacterials and a structure was pursued within SSGCID.

The structures of several acyl-carrier proteins from different organisms have been reported. However, no acyl-carrier protein has been studied from *B. burgdorferi*. In this manuscript, we report almost complete resonance assignment and the high-resolution NMR structure of the acyl-carrier protein from *B. burgdorferi*.

## 2. Methods and experiments

### 2.1. Cloning, protein overexpression and sample preparation

The gene coding for the acyl-carrier protein (UniProt ID O51647; entry name ACP_BORBU) was amplified from the genomic DNA of *B. burgdorferi* using standard PCR techniques. The protein will also be referred to as BobuA.00658.a, its SSGCID identifier. The amplified product was cloned into pET-AVA vector, a modified pET28 vector. The expression construct was transformed into Rosetta *Escherichia coli*. Cells were initially grown at 310 K in M9 minimal medium containing 0.05% ^15^NH_4_Cl and 0.2% ^13^C-glucose (Isotec). After reaching an OD_600_ of 0.4–0.5, the temperature was lowered to 295 K and the cells were induced at an OD_600_ of 0.6–0.7 by the addition of 0.2 m*M* isopropyl β-d-1-thiogalactopyranoside (IPTG) for 16–18 h. The protein was purified using an Ni–NTA column followed by Protease 3C cleavage and gel filtration. The protein eluted as a single peak corresponding to a monomer and was confirmed by SDS–PAGE to be >95% pure. The fractions from the gel filtration containing protein were pooled, concentrated and quantitated by absorption at 280 nm using an absorbance ∊_280_ = 2980 *M* ^−1^ cm^−1^. The final NMR sample contained ∼1.4 m*M* protein, 100 m*M* KCl, 20 m*M* potassium phosphate pH 7.0 in 93% H_2_O plus 7% ^2^H_2_O or in 99.9% ^2^H_2_O for other experiments.

### 2.2. NMR experiments

All NMR experiments were conducted at 298 K on Bruker Avance 500 MHz, Bruker Avance 600 MHz and Varian 800 MHz spectrometers equipped with triple-resonance cryoprobes and pulse field gradients. Experiments recorded on BobuA.00658.a include sensitivity-enhanced 2D [^15^N–^1^H]-HSQC, 3D HNCO, HNCA, HN(CO)CA, CBCACONH, CBCANH, 3D ^15^N-edited TOCSY-HSQC (mixing time 68 ms) and ^15^N/^13^C-edited NOESY-HSQC (mixing times 60 and 120 ms). We also recorded a 2D GFT HNHA (Barnwal *et al.*, 2007 ▶). The data were processed with *NMRPipe* (Delaglio *et al.*, 1995 ▶) and/or *TopSpin* v.2.1 and were analyzed using *CcpNmr* (Vranken *et al.*, 2005 ▶). Proton chemical shifts were calibrated relative to 2,2-dimethyl-2-silapentane-5-sulfonate (DSS) at 298 K (0.000 p.p.m.). Carbon and nitrogen chemical shifts were calibrated indirectly from DSS.

### 2.3. Distance constraints for structure calculations (NOE-derived distance constraints)

Cross-peaks from 3D [^1^H–^1^H]-NOESY-[(^15^N–^1^H)/(^13^C–^1^H)]-HSQC and 2D [^1^H–^1^H]-NOESY spectra were integrated to obtain distance constraints. The calibration of cross-peaks was performed using the macro within *CYANA* with the minimum distance set to 2.4 Å and the maximum distance set to 6.2 Å. Cross-peaks in the NOESY spectra were classified based on their intensities as 1.8–2.7 Å (strong), 1.8–3.7 Å (medium), 1.8–5.0 Å (weak) or 1.8–6.2 Å (very weak). GFT (3,2)D HNHA (Barnwal *et al.*, 2007 ▶) was used to accurately measure ^3^ *J*(H^N^—H^α^). Hydrogen-bond constraints were only added for residues that are involved in α-helices as characterized by initial structures and based on the protection observed in H/D-exchange experiments at a later stage of structure calculation. An upper limit of 2.0 Å was used for the H—O distance in all hydrogen bonds. A total of 1113 distance constraints (an average of ∼15 constraints per residue), which include 345 intraresidue, 308 interresidue (sequential), 245 medium-range and 177 long-range distance constraints and 38 hydrogen-bonding constraints were used in the structure calculations of BobuA.00658.a (Table 1 ▶).

### 2.4. Dihedral angle constraints

Dihedral angle constraints were generated from the measured ^3^ *J*(H^N^—H^α^) (Barnwal *et al.*, 2007 ▶) and by *TALOS* ^+^ (Shen *et al.*, 2009 ▶). A total of 120 (ϕ and ψ) dihedral angle constrains were used in structure calculation (Table 1 ▶).

### 2.5. Structure calculations

Structure calculations were executed using *CYANA*2.1 (Güntert, 2004 ▶). The standard simulated-annealing protocol was used with 10 000 torsion-angle dynamics (TAD) steps. Each round of structure calculations started with 100 randomized conformers. Of all the energy-minimized calculated structures, the 20 structures with the lowest residual target function values were chosen for further analyses. All-atom pairwise r.m.s.d.s were also computed using *CYANA*2.1 (Güntert, 2004 ▶) and *MOLMOL* (Koradi *et al.*, 1996 ▶). The quality of the structures was evaluated using *PROCHECK* (Laskowski *et al.*, 1996 ▶) and the *PSVS* 1.3 web server (Bhattacharya *et al.*, 2007 ▶). The structure was deposited in the PDB under code 2kwl.

## 3. Result and discussion

### 3.1. NMR assignments and data deposition

**Figure 1:**
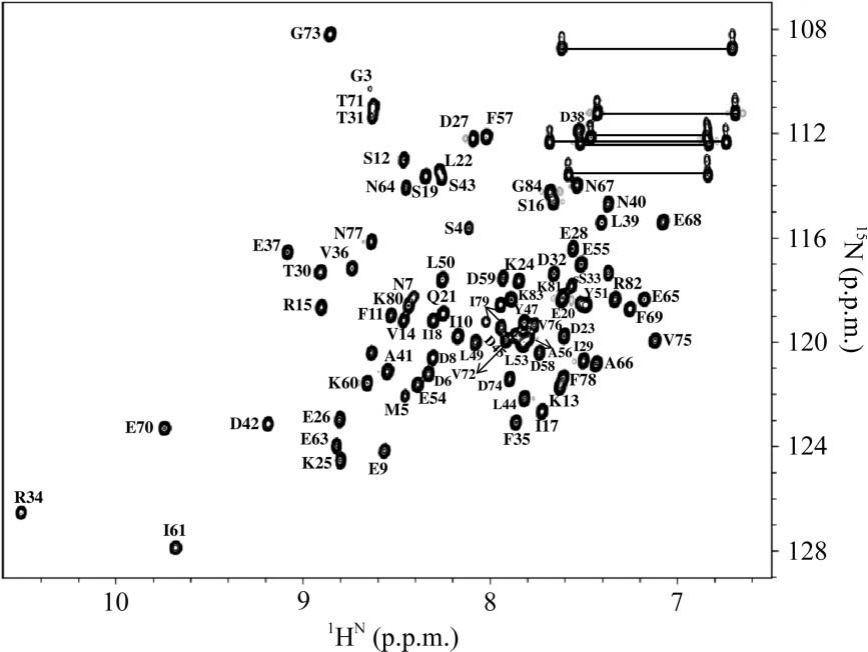
2D [^15^N–^1^H]-HSQC spectrum of BobuA.00658.a recorded at pH 7.0 and 298 K. The spectrum was recorded on a Bruker Avance 600 MHz spectrometer with 1024 and 128 complex points along the *t*_2_ and *t*_1_ dimensions, respectively. The protein concentration was 1.4 m*M* in 93% H_2_O/7% ^2^H_2_O. The peaks are labeled with the single-letter amino-acid code followed by their respective sequence number, as established by sequence-specific assignments of the protein backbone.

The BobuA.00658.a protein is free of Cys, His and Trp residues. We could assign ∼98% of observable backbone resonances and >92% of observable side-chain resonances using triple-resonance experiments as described in §[Sec sec2]2. The ^1^H^N^ and ^15^N resonance assignments for the protein are shown by the single-letter code followed by the sequence number in the [^15^N–^1^H]-HSQC (Fig. 1 ▶). The three NH resonances (Arg34, Ile61 and Glu70) are shifted downfield owing to their involvement in hydrogen bonding. These residues may have a functional role in catalysis, but we have not investigated this role using site-directed mutagenesis. A clearer picture in terms of function and its relation to these residues would require further biochemical experiments. The missing backbone amide resonances consist of two residues in the N-terminal region of the protein which are broadened beyond detection. The information about the ^1^H, ^13^C and ^15^N resonance assignments thus obtained for the protein has been deposited in the BMRB under accession code 16856.

### 3.2. NMR solution structure of BobuA.00658.a

**Figure 2:**
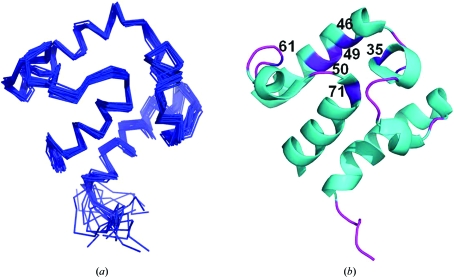
NMR structure of BobuA.00658.a. (*a*) Ensemble of 20 superimposed low-energy NMR-derived structures of the protein (backbone r.m.s.d. = 0.43 ± 0.11 Å) in ribbon representation. (*b*) Cartoon representation; important residues in the hydrophobic core are shown in purple color with their respective sequence number. Images were generated using *PyMOL* (http://www.pymol.org).

The NMR structure of BobuA.00658.a was determined using distance constraints and dihedral constraints as detailed in Table 1 ▶. Fig. 2 ▶ shows the ensemble of 20 superimposed structures derived using *CYANA*. The quality of the structure was verified with *PROCHECK* (Laskowski *et al.*, 1996 ▶), which revealed that none of the residues lie in disallowed regions of the Ramachandran plot. The structural and Ramachandran statistics for BobuA.00658.a are also provided in Table 1 ▶. The polypeptide segments consisting of residues 7–22, 34–37, 43–57 and 72–83 form four α-helices, whereas segments 26–28 and 65–68 form 3_10_-helices. Within the inner face of the helices, several hydrophobic side chains form the core of the protein (Fig. 2 ▶ *b*).

**Figure 3:**
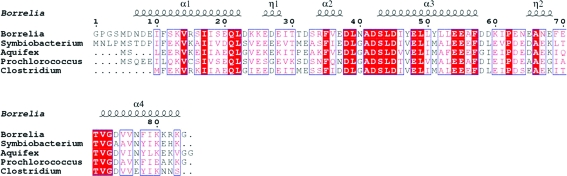
Sequence alignment of the acyl-carrier protein from *B. burgdorferi* compared with related acyl-carrier proteins from different prokaryotic organisms: *S. thermophilum*, *A. aeolicus*, *P. marinus* and *C. thermocellum*. The alignment was produced using *ESPript* 2.2 (http://espript.ibcp.fr/ESPript/ESPript/). Residues with high sequence similarity and identity are shown in closed boxes and as colored regions, respectively. The secondary structures from PDB entry 2kwl are shown at the top of the figure.

Sequence alignment with the enzymes from *Symbiobacterium thermophilum*, *Aquifex aeolicus*, *Prochlorococcus marinus* and *Clostridium thermocellum* revealed that BobuA.00658.a has 48% sequence similarity to the enzyme from *S. thermophilum*, 44% to that from *A. aeolicus*, 52% to that from *P. marinus* and 58% to that from *C. thermocellum* (Fig. 3 ▶). A structural homology search using the *DALI* server (http://ekhidna.biocenter.helsinki.fi/dali_server) revealed that this protein has 37% similarity to the acyl-carrier protein from *A. aeolicus*, which is structurally closest to it. The backbone r.m.s.d. between the structures from *A. aeolicus* and *B. burgdorferi* was 3.1 Å, whereas alignments of BobuA.00658.a with structures from other organisms had higher values. Only residues 8–82 of BobuA.00658.a were selected for r.m.s.d. comparison. Based on the UniProt and homology search, Ser39 seems to be a central residue involved in fatty-acid binding. This residue is close to the core involving three downfield-shifted residues (Arg34, Ile61 and Glu70). Finally, a proper study including mutation *in vivo* will provide a clearer picture regarding fatty-acid binding.

## 4. Conclusions

We report here the structure of an acyl-carrier protein from *B. burgdorferi*. Since this protein was selected as a potential target for drug-discovery efforts (Myler *et al.*, 2009 ▶; Younger & Orsher, 2010 ▶) owing to its involvement in fatty-acid biosynthesis, its structural and dynamic features could be used to better understand its acyl-carrier activity and to discover inhibitors of its essential function. This information could be of value in discovering small-molecule inhibitors for this activity, which could be used in the treatment of Lyme disease.

## Supplementary Material

PDB reference: BobuA.00658.a, 2kwl

## Figures and Tables

**Table:** Residues 8–82 were used in the Ramachandran statistics as the N-terminal residues are unstructured.

NMR distance and dihedral constraints	
Distance constraints	
Total NOE	1113
Intraresidue (|*i* − *j*| = 0)	345
Interresidue	
Sequential (|*i* − *j*| = 1)	308
Medium range (1 < |*i* − *j*| < 5)	245
Long range (|*i* − *j*| ≥ 5)	177
Other (hydrogen-bond constraints)	38
Dihedral angle constraints	
ϕ (°)	60
ψ (°)	60
Constraint violations	
NOE distance violations (>0.1 Å)	None
van der Waals violations (>0.1 Å)	1
Dihedral angle violations (>3°)	None
Average target function (Å^2^)	1.28 ± 0.09
Average r.m.s. deviation (Å)	
Backbone atom (8–82)	0.42 ± 0.11
Heavy atom (8–82)	0.93 ± 0.13
Ramachandran plot statistics (%)	
Residues in most favored regions	81.0
Residues in additional allowed regions	16.9
Residues in generously allowed regions	2.1
Residues in disallowed regions	0.0

**Table:** Structure-quality factors: overall statistics.

	Mean score	SD	*Z* score†
*PROCHECK G* factor‡ (ϕ, ψ only)	−0.36	N/A	−1.10
*PROCHECK G* factor‡ (all dihedral angles)	−0.66	N/A	−3.90
*Verify*3*D*	0.19	0.0215	−4.33
*ProSA* II (−ve)	0.24	0.0483	−1.70
*MolProbity* clash score	30.66	3.0089	−3.74

† With respect to mean and standard deviation for for a set of 252 X-­ray structures of <500 residues with resolution ≤ 1.80 Å, *R* factor ≤ 0.25 and *R*_free_ ≤ 0.28; a positive value indicates a ‘better’ score. (Generated using *PSVS* 1.3.)

‡ Residues with sum of ϕ and ψ order parameters >1.8. Ordered residue range 7–83. (Generated using *PSVS* 1.3.)
